# Unconventional Extraction and Storage Strategies in Order to Enhance the Shelf Life of Virgin Olive Oil

**DOI:** 10.3390/foods13132088

**Published:** 2024-07-01

**Authors:** Monica Macaluso, Nicola Mercanti, Ylenia Pieracci, Roberto Mangia, Piero Giorgio Verdini, Angela Zinnai

**Affiliations:** 1 Department of Agriculture, Food and Environment, University of Pisa, Via del Borghetto 80, 56124 Pisa, Italy; monica.macaluso@unipi.it (M.M.); nicola.mercanti@phd.unipi.it (N.M.); r.mangia@studenti.unipi.it (R.M.); angela.zinnai@unipi.it (A.Z.); 2 Department of Pharmacy, University of Pisa, Via Bonanno 6, 56126 Pisa, Italy; 3 European Organization for Nuclear Research, Espl. des Particules 1, 1211 Meyrin, Switzerland; piero.giorgio.verdini@cern.ch; 4 Interdepartmental Research Centre “Nutraceuticals and Food for Health”, University of Pisa, Via del Borghetto 80, 56124 Pisa, Italy

**Keywords:** virgin olive oil, extraction, storage, packaging, phenols

## Abstract

Virgin olive oil (VOO) is a globally esteemed product renowned for its chemical composition, nutritional value, and health benefits. Consumers seeking natural, nutritious, and healthy foods increasingly favor VOO. The optimization of the extraction system ensures the production of high-quality VOO with abundant antioxidant compounds that naturally protect it from degradation. Proper storage is crucial in maintaining the quality of VOO, prompting the exploration of novel extraction and preservation techniques. Factors such as light, temperature, and oxygen greatly influence the degradation process, resulting in reduced levels of natural antioxidants like polyphenols. Undesirable by-products and non-aromatic compounds may be formed, making the oil unacceptable over time. On the basis of all this consideration, this study aimed to evaluate the synergic use of two different gases (CO_2_ and argon) during the malaxation phase to limit radical development and delay lipid autoxidation. Additionally, unconventional preservation systems, namely argon headspace, shellac, and bottle in bag, were assessed over a period of 150 days. The results evidenced that the use of CO_2_ and argon during the malaxation process resulted in an improvement in the oil quality compared to the one obtained with the traditional system. However, in traditional oils, the alternative packaging systems determined interesting outcomes as they were able to positively affect different parameters, while the packaging effect was more mitigated in the test oils.

## 1. Introduction

Virgin olive oil (VOO) is widely regarded as a premium oil due to several factors, including its advanced physical extraction technology, high monounsaturated fatty acid composition, abundant minor bioactive compounds, and exceptional sensory attributes [1,2,3]. The malaxation phase is crucial as it significantly influences the quality and yield of the oil. Traditionally, this process is conducted in the presence of air, but recent advancements have explored the use of different gases to improve the quality and stability of the final product [4]. Oxygen is a double-edged sword in olive oil production. On the one hand, a certain amount of oxygen is necessary during malaxation to enhance the enzymatic activities that improve the sensory characteristics of the oil, such as flavor and aroma. Lipoxygenase and other oxidative enzymes require oxygen to function, and these enzymes contribute to the formation of volatile compounds that define the fresh, fruity notes of high-quality virgin olive oil [5]. However, excessive exposure to oxygen can lead to oxidation of the oil, resulting in the degradation of beneficial phenolic compounds and the formation of off-flavors. Oxidation also negatively impacts the oil’s shelf life, as it accelerates the rancidity process [4]. To mitigate the adverse effects of oxygen, the use of inert gases like nitrogen (N_2_) and argon (Ar) during the malaxation phase has been investigated [4]. These gases can create an anaerobic environment that prevents oxidation. Carbon dioxide (CO_2_) has also been explored as an alternative to oxygen during malaxation [6]. Using CO_2_ gas can create a protective atmosphere that minimizes oxidation, similar to nitrogen and argon. It has been found to maintain the phenolic content and sensory attributes of the oil, though its efficacy is generally considered comparable to that of nitrogen. However, the progress of oxidation is influenced by various factors, including exposure to oxygen, light, high temperatures, and the presence of pro-oxidant or antioxidant compounds within the oil [7]. In this regard, selecting an appropriate container becomes vital as it directly regulates the oil’s exposure to oxygen and light. Glass, metals, and plastics are commonly used materials for VOO packaging. Recent studies have investigated the performance of these packaging materials under different storage conditions, revealing their respective advantages and disadvantages [8,9,10,11,12]. For instance, dark glass bottles offer excellent protection against oxygen and light, in addition to being environmentally friendly and easy to clean, sterilize, and reuse [13]. Further evidence suggests that packaging VOO in glass bottles with a nitrogen atmosphere can significantly prolong its shelf life [14]. Plastic containers possess several advantages, including suitable mechanical properties, resistance to damage, chemical inertness, low oxygen permeability, affordability, and recyclability [15,16]. However, Kanavouras demonstrated that PET properties, such as oxygen transmission rates and humidity sorption, undergo changes during olive oil storage [17]. Additionally, PET bottles can be transformed into active packaging by incorporating UV adsorbent pigments or oxygen scavengers like hydroxytyrosol, which aid in preventing light penetration and blocking oxidation reactions. Some studies have also reported that olive oil can penetrate polyethylene (PE) packaging, causing a plasticizing effect on the polymers and swelling [17]. These studies highlight a strong change that has occurred in the olive oil storage landscape, forcing production companies to review the means used to date. Given these considerations, this study aims to address the critical issue of VOO degradation by investigating the use of two different gases, CO_2_ and argon, during the malaxation phase to limit radical development and delay lipid autoxidation. Furthermore, the research evaluates unconventional preservation systems, such as argon headspace, shellac, and bottle in bag, over a period of 150 days. The objective is to identify innovative extraction and preservation methods that enable the production of “long-lasting” oil, which remains consumable beyond 12 months from production while maintaining its chemical qualities stable. By aligning the extraction and storage strategies with the unique properties of VOO, this work seeks to enhance its shelf life and preserve its nutritional and sensory attributes.

## 2. Materials and Methods

The olive oils were obtained using the fruits of *Olea europaea* variety “Frantoio”, harvested during the 2022/2023 season crop by the Rossi Piero company, Massa Marittima (GR).

A total of 200 kg of olives were harvested from twenty olive trees using picking combs. The olives were homogenized before processing and divided into 6 batches of 33 kg each (with three extraction runs for each trial) in order to ensure consistency among the samples.

The extraction runs were performed using a micro oil mill equipped with a two-phase decanter (Spremioliva C30, Toscana Enologica Mori, Tavernelle Val Di Pesa, Florence, Italy). This advanced mill exhibited a milling capacity of 25–35 kg of olives per hour, and the extraction process followed the protocol previously described [18]. For the malaxation process, the machine was modified with hermetic closures and dedicated valves for gas injection, which allowed precise control of the gas composition. The employed gases were CO_2_ and argon with a purity of 99.99%, supplied by SIAD spa, Italy. Additionally, the machine was equipped with a thermal control system to maintain a constant temperature of 22–25 °C during the process. The virgin olive oils (VOOs) obtained underwent initial homogenization in specialized stainless steel tanks and were subsequently stored in 100 mL greenish glass bottles. The control samples were obtained in the same oil mill but using air for the malaxation process, while for test samples, the malaxation process was performed under a modified atmosphere of CO_2_ and argon_._ The employed gas showed a purity of 99.99% and was supplied by SIAD spa (Italy). Both test and control samples were then subjected to different storage conditions, obtaining the samples reported in Table 1.

Samples were analyzed after 30, 90, and 150 days for chemical characterization, for a total of five months, considering the limited amounts of the obtained product. For each malaxation/packaging combination, two bottles were sampled.

### 2.1. Raw Material Determination

The samples were characterized according to the fruit classification described in the method [19].

### 2.2. Legal Quality Parameters

According to the EU Regulation 2022/2014 [20], free fatty acidity (FFA) and the peroxide value (PV) determinations, as well as the specific absorbances at 232 and 270 nm (K232, K270, and ΔK), of the ROOs were carried out in duplicate.

### 2.3. Total Phenol Determination

Total phenols were extracted following the procedure of Servili et al. [21] with slight modifications [10]. Extracts were stored at −20 °C under N_2_ atmosphere until analyses. The total phenol concentration of the VOOs was determined calorimetrically at 765 nm using the Folin–Ciocalteau reagent [9]. Calculations were carried out using a calibration curve with gallic acid as a standard.

### 2.4. Bitterness Intensity Determination

Bitter components were extracted from 1.00 ± 0.01 g of oil samples using 6 mL extraction columns (Sep-Pak C18 Classic Cartridge, Waters s.p.a., Sesto San Giovanni (MI), Italy), and the IB was determined following the method previously described [22] recording the absorbance at 225 nm.

### 2.5. Chlorophylls and Carotenoids Determination

Chlorophylls and carotenoids were determined at 670 nm and 470 nm, respectively, following Minguez-Mosquera et al.’s [23] protocol. The oil samples were dissolved in cyclohexane (1.5:5 *w*/*v*), and absorbance was measured using a Perkin Helmer Lambda 10 UV–vis spectrophotometer. Chlorophylls and carotenoids contents were determined as follows:

Chlorophylls = (A670 × 106)/(613 × 100 × d)(1)

Carotenoids = (A470 × 106)/(2000 × 100 × d)(2)

where *A* is the absorbance and *d* is the path length of the cell (1 cm).

### 2.6. Antioxidant Activity Determination

The antioxidant capacity of the phenolic extracts of the EVOO samples was assessed by ABTS assay, according to the procedure reported by Sgherri et al. [24]. The radical solution was prepared as described by Fellegrini et al. [25], and a Trolox dose–response curve in the 0.2–1.5 mmol range was used. The antioxidant activity was expressed as Trolox equivalent antioxidant capacity (TEAC) mL^−1^ extract.

### 2.7. Statistical Analysis

Results are expressed as means ± SD (n = 3). The significance of differences between the different storage periods and the diverse packaging conditions was determined by one-way ANOVA using Tukey’s post hoc test to separate averages. Two-way ANOVA was further performed to evaluate the influence of the malaxation process, packaging conditions, and their interaction on the investigated parameters. For both analyses, a *p* < 0.05 was used to assess the significance of differences between means. Finally, multivariate statistical analysis with the Principal Component Analysis (PCA) method was performed on a correlation data matrix of 32 × 7 (32 samples × 7 parameters = 224 data), selecting the two highest PCs obtained by the linear regressions: the chosen PC1 and PC2 covered 73.2% and 17.0% of the variance, respectively, for a total explained variance of 90.2%. Statistical analyses were carried out using the JMP Pro 14.0.0 software package (SAS Institute, Cary, NC, USA).

## 3. Results

### 3.1. Olive Fruits Characterization

The olive fruits used for the production of the oils object of the present study showed the characters reported in Table 2. A rather high maturity index can be noted due to a particularly unusual production year characterized by an unstable climate and uncommon atmospheric events. These data have a great influence on the final product.

### 3.2. Olive Oil Quality Characters at Starting Time

At the beginning of the experimentation, immediately after the extraction process, the oils produced were, therefore, below the parameters expected for an extra virgin olive oil. From the point of view of the parameters expected for the production classification, the oils were within the parameters expected for a virgin olive oil (Reg. 2022/2014) [20]. From a qualitative point of view, they were characterized by fairly high acidity and peroxides and quite low total phenols, intensity of bitterness, and antioxidant power (Table 3).

### 3.3. Free Fatty Acidity Trend

The free acidity in control and test oils, besides being significantly different immediately after the milling (T0), also showed a different trend during the storage. Indeed, control oil is no longer suitable for human consumption unless rectified (Reg. 2022/2014), already after 30 days of storage, showing an increase of 49.23% after 150 days. Conversely, although a significant increase during the storage has also been registered for test oils (18.96% after 150 days), their free acidity remained below the legally accepted threshold level for virgin olive oils (2%) until the third month (90 days) and slightly exceeded it after 150 days. As evidenced by the two-way analysis of variance (Table 4), free acidity was strongly influenced by the malaxation process. Conversely, packaging conditions did not determine significant variation in this parameter for the first month of storage, which conversely resulted in a significantly positive influence from 90 days. In particular, innovative packaging systems have better preserved the product in terms of free acidity compared to the traditional air-headspace packaging, and it was evident mainly for control oil. In fact, control oil with argon headspace showed a free acidity lower of 6% over 150 days of storage, “bottle in bag” oil of 17%, and shellac wax oil of 20%. Conversely, the increase in the free acidity, over the fifth month of storage, in test oils produced with the innovative malaxation process was more contained if compared to the traditional ones, and in this case, the different packaging systems did not show a significant influence.

Thus, the obtained results evidenced that the free acidity increase during the storage period was more marked in the control oils, for which a greater packaging effect was noticed. The innovative malaxation process seemed to allow us to obtain a product characterized by both a better state of preservation suddenly after the oil milling and greater durability during storage, proving to be a proper method for prolonging oil shelf life.

### 3.4. Peroxide Values and UV Spectrophotometric Indices

Peroxide concentrations of the analyzed samples are reported in Table 5.

Both control and test oils, packed in the different alternative conditions, had a peroxide concentration below the acceptability legal threshold fixed at 20 ppm. During the storage period, peroxide concentration showed a trend similar to that reported in the literature [26]. Indeed, their content increased during the former three months, reached a maximum, and then shrunk again. This evolution was plausible and could be related to the secondary oxidative phase of lipid autoxidation, during which peroxides are converted to carbonilic volatile compounds [26]. Both the malaxation process and package system seemed to significantly influence the progression of peroxides during product storage. In detail, the innovative malaxation process allowed us to obtain initial oils with a lower peroxides content. However, while the test samples packed with air headspace and argon headspace, similarly to all the control oils, showed the peculiar bell-shaped trend of peroxides during storage, the ones packed with shellac wax and “bottle in bag” evidenced a more linear progression, suggesting slower autoxidation reactions.

Although the peroxides trend rate was unusual and could be attributed to the initialization of the autoxidative reaction in the olive fruits, whose phytosanitary conditions were not optimal, the K_232_ value (Table 6), index of primary oxidation products [27], exceeded the legal threshold of 2.6 only at the last month of storage in the control oil with air headspace. All the other samples, both controls with different package conditions and test samples, maintained the parameter within the allowed limit. Furthermore, the K_270_ index (Table 6), related to the secondary oxidation process, and, in particular, to the conjugated trienes and carbonyl compounds contents, resulted in lower than the legal limit of 0.25 in all the tested samples, both control and test, during all the storage periods and in every packaging conditions. The Δ*K* value, corresponding to the variation within the 270 nm region, was within the allowed legal amount of 0.01 in all the test oil for all the storage period, while control ones showed a value closer to the aforementioned limit, sometimes exceeding it, especially during the last months of storage.

Considering the effect of the packaging on this parameter, it was particularly evident for the oils produced with the traditional malaxation process, in which a reduction of 9% was noticed for argon-headspace oil, 6% for “bottle in bag” oil, and 19% for shellac oil, if compared to the air-headspace one. Conversely, for the samples obtained with the innovative malaxation process, no significant differences among the different package systems were found.

### 3.5. Biochemical Analyses

Phenolic compounds represent important bioactive molecules of virgin olive oil, responsible for its pungent taste and for its great stability [28]. At the initial stage (Table 7), the total phenolic content was higher in the control than in the test oil, probably due to the greater oleuropein content, evidenced by the higher bitter intensity value [29]. Nevertheless, although the higher initial concentration. Polyphenols showed a greater degradation rate during the conservation period in the control oil stored in all the different conditions compared to the analogous test samples, evidencing the influence of the different malaxation processes. As previously introduced, one of the major phenolic compounds of olive oil is oleuropein, which, besides being responsible for positive effects on human health, imparts the characteristic bitter taste to the product [30]. The bitter intensity, similarly to total phenolic content, showed a reduction during the storage period, probably related to the progressive oxidation of oleuropein, and even in this case, the reduction was more marked in the control oils. The positive effect of the packaging on the bitter index was more evident in the traditional oil, as the different package systems were associated with a lower reduction in this parameter and, thus, a greater preservation of oleuropein. Conversely, in the oils produced by the alternative malaxation process, lower differences in the bitter index value among the different package systems were revealed.

Despite the greater initial phenolic content in the control oils, those samples showed an antioxidant potential, expressed as µmol di TEAC/mL of extract (Table 7), similar to the test samples analogously packed, and even in this case, the decrease during the storage period was more marked in control than in test oils (reduction of 78% in control oils and 65% in test oils), similarly to the phenolic trend, evidencing the strong correlation between those parameters.

Carotenoids, as well as chlorophyll contents, showed a reduction during the storage period in both control and test samples (Table 8) without significant differences among the different packaging conditions. However, while no differences were revealed between the analogous samples of control and test oils for the carotenoids, chlorophylls tended to be higher in the oils produced with the innovative malaxation process.

### 3.6. Statistical Analysis

**Figure 1:**
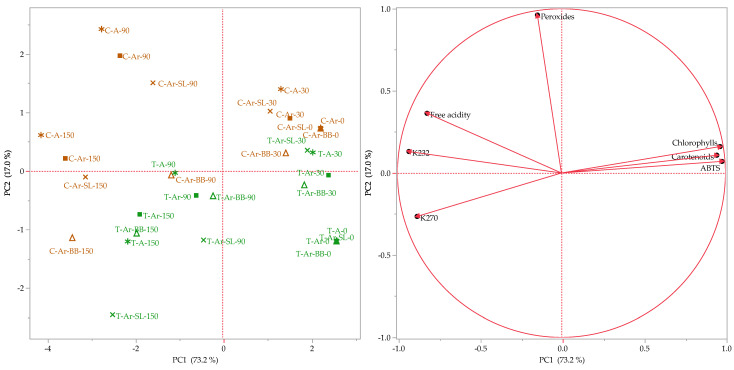
Score (**left**) and loading (**right**) plots of the Principal Component Analysis performed on the investigated parameters of the analyzed samples.

The results of the analyses performed on the oils produced with the two different malaxation protocols and packed in different conditions were subjected to multivariate statistical analysis with the Principal Component Analysis method. The score plot (Figure 1) evidenced a clear separation of the oils according to the malaxation process since the control and the test samples, colored in red and blue, respectively, seemed separated by a diagonal line, and the former was almost entirely plotted in the upper quadrants, while the latter in the bottom ones. This division was probably attributable to the peroxide content, whose vector was directed toward the upper part of the upper quadrants of the loading plot, explained by the fact that, actually, oils produced with the traditional malaxation protocol were characterized by a higher concentration of peroxides.

Nevertheless, a gathering of the samples according to the storage time was also visible. In fact, it was possible to see that oils at T0 and T1 were plotted in the rightest part of the score plot, and as the storage time increased, they were shifted more to the left, as pigments content and ABTS value, whose vectors were directed to the rightmost part of the loading plots, decreases, while the spectrophotometric indices and the free acidity increased.

Although the statistical treatment evidenced a stronger influence of the malaxation process and of the storage time on the quality parameters of the analyzed oils, it was also possible to notice that the effect of the different packaging conditions increased with the increase in the storage period. Indeed, the oils analyzed after one month of storage, both control and test, packed in different conditions, were plotted very closely in the score plot. Instead, the ones analyzed after both three and five months showed more discrepancies. In particular, for both times, the alternative system, bottle in bag, seemed to be the most advantageous, as the samples packed with this method were plotted closer to the test oils.

The results of this study show that the use of inert gases such as CO_2_ and argon during malaxation, in combination with innovative preservation systems such as “bottle-in-bag,” can significantly improve the quality of extra virgin olive oil (VOO) by reducing oxidation and prolonging its shelf life.

The effectiveness of inert gases in reducing oxidation during VOO production has already been highlighted. For example, previous studies have shown that the use of CO_2_ can reduce peroxide levels and improve the oxidative stability of VOO. The results of this work confirm these observations, indicating that the use of CO_2_ during malaxation significantly reduces volatile acidity and peroxide values compared to the control. However, this study goes further, showing that the use of argon can be even more effective, something that has not been extensively explored in the existing literature.

## 4. Conclusions

The results evidenced that the use of CO_2_ and argon during the malaxation process determined an improvement in the oil quality compared to the one obtained with the traditional system. Besides a greater initial quality, the products obtained with the innovative system also showed improved preservation. The degradation processes, indeed, follow reaction kinetics differently according to the applied malaxation procedure. With reference to free fatty acidity control oils, results were no longer suitable for human consumption after 30 days of storage, while for test oils, the parameters exceeded the law threshold level after 90 days of conservation. All samples underwent a degradation of the phenolic fraction, which was also evidenced by the degradation of oleuropein and by the reduction in the antioxidant potential highlighted in all the samples but more contained in the ones obtained with the innovative malaxation procedure. The effect of the packaging system on parameters like free acidity, K232, and bitter intensity was also evident in the control oil, while it was more mitigated in the test ones. The other investigated parameter, instead, did not vary significantly according to the packaging in both control and test oils. Future research could focus on the long-term analysis of VOO preservation using these innovative methods, extending the shelf life beyond 180 days. Furthermore, it would be interesting to explore the effect of other gas atmospheres and advanced preservation systems to further optimize VOO quality. Finally, large-scale comparative studies could help validate the results obtained and strengthen recommendations for olive oil producers.

## Figures and Tables

**Table 1:** Samples coding and description.

Samples	Description
C-A—Control	Oil obtained by the classical malaxation process and packed with air headspace.
C-Ar—Control with Argon HS	Oil obtained by the classical malaxation process and packed with argon headspace.
C-Ar-BB—Control “bottle in bag” with Argon HS	Oil obtained by the classical malaxation process and packed with the “bottle in bag” protocol with argon headspace in the bag.
C-Ar-SL—Control shellac with Argon HS	Oil obtained by the classical malaxation process and packed with argon headspace and waxy cap.
T-A—Test	Oil obtained by the alternative malaxation process and packed with air headspace.
T-Ar—Test with Argon HS	Oil obtained by the alternative malaxation process and packed with argon headspace.
T-Ar-BB—Test “bottle in bag” with Argon HS	Oil obtained by the alternative malaxation process and packed with the “bottle in bag” protocol with argon headspace in the bag.
T-Ar-SL—Test shellac with Argon HS	Oil obtained by the alternative malaxation process and packed with argon headspace and waxy cap.

**Table 2:** Olive fruit characterization. Data are expressed as mean ± confidence interval (n = 3) at *p* = 0.05.

Parameter	Values
Maturity Index (0:7)	4.00 ± 0.10
Dry Matter (%)	47.50 ± 0.03
Oil Content (% d.m.)	21.50 ± 0.04

**Table 3:** Chemical characterization of the two produced oils (control and test).

Parameters	Control	Test	Legal Thresholds for Virgin Olive Oils
Free acidity (g oleic acid/100 g oil)	1.94 ± 0.03 ^A^	1.78 ± 0.05 ^B^	<2.00
Peroxides (meq O_2_/kg oil)	13.90 ± 0.20 ^A^	9.95 ± 0.10 ^B^	<20.00
K232	1.77 ± 0.02 ^A^	1.56 ± 0.00 ^B^	<2.6
K270	0.10 ± 0.01	0.09 ± 0.00	<0.25
ΔK	0.001 ± 0.000	0.001 ± 0.000	<0.01
Intensity of bitterness (BI)	1.04 ± 0.08 ^A^	1.40 ± 0.08 ^A^	-
Total phenols (mg gallic acid/kg oil)	327.61 ± 1.66 ^A^	299.38 ± 3.27 ^B^	-
Antioxidant power (µm TEAC/mL extract)	0.23 ± 0.04	0.23 ± 0.05	-
Chlorofills (ppm of pheophytin)	6.36 ± 0.06 ^A^	5.77 ± 0.18 ^B^	-
Carotenoids (ppm of lutein)	3.10 ± 0.07	3.04 ± 0.01	-

The letters highlighted significant differences between the different kneading conditions of the control and test samples after ANOVA one-way analysis.

**Table 4:** (**A**) Free acidity progression during the storage of the control and the test oil packaged in alternative conditions. (**B**) Two-way analysis of variance performed to evaluate the influence of the malaxation process, packaging conditions, and their interaction on the free acidity of the samples during the storage.

**(A) Samples**	**Free Acidity (** **g Oleic Acid/100 g Oil)**			
	**D0**	**D30**	**D90**	**D150**
C-A	1.96 ± 0.02 ^D^	2.10 ± 0.01 ^a,C^	2.93 ± 0.03 ^a,B^	3.12 ± 0.01 ^a,A^
C-Ar	1.96 ± 0.02 ^C^	2.02 ± 0.00 ^c,C^	2.75 ± 0.04 ^a,B^	3.01 ± 0.01 ^b,A^
C-Ar-BB	1.96 ± 0.02 ^C^	2.06 ± 0.00 ^b,B^	2.80 ± 0.01 ^a,A^	2.83 ± 0.01 ^c,A^
C-Ar-SL	1.96 ± 0.02 ^C^	2.09 ± 0.00 ^ab,C^	2.43 ± 0.14 ^b,B^	2.74 ± 0.02 ^d,A^
T-A	1.78 ± 0.00 ^B^	1.89 ± 0.08 ^B^	2.06 ± 0.01 ^a,A^	2.11 ± 0.01 ^A^
T-Ar	1.78 ± 0.00 ^C^	1.87 ± 0.03 ^B^	1.97 ± 0.02 ^b,A^	2.03 ± 0.03 ^A^
T-Ar-BB	1.78 ± 0.00 ^C^	1.92 ± 0.01 ^B^	2.07 ± 0.01 ^a,A^	2.08 ± 0.01 ^A^
T-Ar-SL	1.78 ± 0.00 ^C^	1.86 ± 0.01 ^B^	2.03 ± 0.03 ^ab,A^	2.05 ± 0.02 ^A^
**(B) Storage Period**	**Malaxation Process**	**Packaging Conditions**	**Malaxation Process × Packaging Conditions**	
D0	***	n.s.	n.s.	
D30	***	n.s.	n.s.	
D90	***	**	**	
D150	***	***	***	

Lowercase upper letters evidenced significant differences among the different packaging conditions of control and test samples. Uppercase upper letters evidenced significant differences among the different storage times. LSR, ** *p* < 0.01; *** *p* < 0.0001; n.s.: not significative.

**Table 5:** (**A**) Peroxide progression during the storage of the control and the test oil packaged in alternative conditions. (**B**) Two-way analysis of variance performed to evaluate the influence of the malaxation process, packaging conditions, and their interaction on the peroxide concentration of the samples during the storage.

**(A) Samples**	**Peroxide Value (meq O_2_/kg of Oil)**			
	**D0**	**D30**	**D90**	**D150**
C-A	13.90 ± 0.14 ^C^	15.43 ± 0.12 ^a,B^	17.54 ± 0.68 ^a,A^	13.69 ± 0.04 ^a,C^
C-Ar	13.90 ± 0.14 ^BC^	14.55 ± 0.46 ^a,B^	16.84 ± 0.56 ^a,A^	13.0 ± 0.14 ^b,C^
C-Ar-BB	13.90 ± 0.14 ^A^	13.15 ± 0.02 ^b,B^	12.15 ± 0.21 ^b,C^	10.15 ± 0.21 ^c, D^
C-Ar-SL	13.90 ± 0.14 ^C^	14.69 ± 0.09 ^a,B^	16.38 ± 0.13 ^a,A^	13.05 ± 0.13 ^b,D^
T-A	9.95 ± 0.07 ^C^	13.35 ± 0.12 ^a,A^	13.48 ± 0.21 ^a,A^	11.49 ± 0.07 ^b,B^
T-Ar	9.95 ± 0.07 ^B^	12.28 ± 0.09 ^b,A^	12.71 ± 0.04 ^b,A^	12.51 ± 0.31 ^a,A^
T-Ar-BB	9.95 ± 0.07 ^C^	12.11 ± 0.23 ^b,AB^	12.62 ± 0.07 ^b,A^	11.68 ± 0.28 ^ab,B^
T-Ar-SL	9.95 ± 0.07 ^C^	13.51 ± 0.39 ^a,A^	10.95 ± 0.13 ^c,B^	9.67 ± 0.07 ^c,C^
**(B) Peroxides**	**Malaxation Process**	**Packaging Conditions**	**Malaxation Process × Packaging Conditions**	
D0	***	n.s.	n.s.	
D30	***	***	*	
D90	***	***	***	
D150	***	***	***	

Lowercase upper letters evidenced significant differences among the different packaging conditions of control and test samples. Uppercase upper letters evidenced significant differences among the different storage times. LSR, * *p* < 0.05; *** *p* < 0.0001; n.s.: not significative.

**Table 6:** (**A**) Spectrophotometric indices progression during the storage of the control and the test oil packaged in alternative conditions. (**B**) Two-way analysis of variance performed to evaluate the influence of the malaxation process, packaging conditions, and their interaction on the spectrophotometric indices of the samples during the storage.

**(A) Samples**	**K232**				**K270**			
	**D0**	**D30**	**D90**	**D150**	**D0**	**D30**	**D90**	**D150**
C-A	1.77 ± 0.01 ^D^	1.87 ± 0.01 ^b,C^	2.32 ± 0.01 ^a,B^	2.57 ± 0.03 ^a,A^	0.10 ± 0.01 ^D^	0.1 ± 0.00 ^a,C^	0.16 ± 0.01 ^a,B^	0.22 ± 0.00 ^a,A^
C-Ar	1.77 ± 0.01 ^D^	1.85 ± 0.04 ^b,C^	2.30 ± 0.02 ^a,B^	2.40 ± 0.02 ^c,A^	0.10 ± 0.01 ^C^	0.12 ± 0.01 ^b,C^	0.17 ± 0.00 ^a,B^	0.21 ± 0.01 ^b,A^
C-Ar-BB	1.77 ± 0.01 ^C^	1.86 ± 0.05 ^b,B^	1.79 ± 0.01 ^b,C^	2.46 ± 0.01 ^b,A^	0.10 ± 0.01 ^C^	0.12 ± 0.01 ^b,C^	0.13 ± 0.01 ^b,B^	0.21 ± 0.01 ^b,A^
C-Ar-SL	1.77 ± 0.01 ^D^	1.96 ± 0.00 ^a,C^	2.07 ± 0.03 ^c,B^	2.23 ± 0.00 ^d,A^	0.10 ± 0.01 ^C^	0.12 ± 0.01 ^ab,C^	0.14 ± 0.01 ^b,B^	0.22 ± 0.01 ^a,A^
T-A	1.65 ± 0.00 ^D^	1.78 ± 0.01 ^C^	2.10 ± 0.04 ^a,B^	2.21 ± 0.00 ^A^	0.09 ± 0.00 ^C^	0.11 ± 0.01 ^B^	0.15 ± 0.01 ^a,B^	0.21 ± 0.01 ^a,A^
T-Ar	1.65 ± 0.00 ^C^	1.77 ± 0.04 ^C^	1.99 ± 0.01 ^b,B^	2.21 ± 0.09 ^A^	0.09 ± 0.00 ^C^	0.10 ± 0.00 ^C^	0.14 ± 0.00 ^ab,B^	0.20 ± 0.01 ^ab,A^
T-Ar-BB	1.65 ± 0.00 ^C^	1.77 ± 0.06 ^B^	1.73 ± 0.03 ^d,B^	2.22 ± 0.00 ^A^	0.09 ± 0.00 ^C^	0.11 ± 0.01 ^C^	0.12 ± 0.01 ^c,B^	0.20 ± 0.01 ^ab,A^
Ts	1.65 ± 0.00 ^D^	1.76 ± 0.08 ^C^	1.86 ± 0.00 ^c,B^	2.15 ± 0.01 ^A^	0.09 ± 0.00 ^C^	0.10 ± 0.00 ^C^	0.13 ± 0.00 ^ab,B^	0.30 ± 0.00 ^b,A^
**(B) K232/K270**	**Malaxation Process**			**Packaging Conditions**		**Malaxation Process × Packaging Conditions**		
D0	***/**			n.s./n.s.		n.s./n.s.		
D30	***/***			*/***		**/*		
D90	***/***			***/***		***/*		
D150	***/***			***/**		***/n.s.		

Lowercase upper letters evidenced significant differences among the different packaging conditions of control and test samples. Uppercase upper letters evidenced significant differences among the different storage times. LSR, * *p* < 0.05; ** *p* < 0.01; *** *p* < 0.0001; n.s.: not significative.

**Table 7:** (**A**) Phenol content and ABTS value progressions during the storage of the control and the test oil packaged in alternative conditions. (**B**) Two-way analysis of variance performed to evaluate the influence of the malaxation process, packaging conditions, and their interaction on phenols concentration and ABTS value of the samples during the storage.

**(A) Samples**	**Total Phenols**				**ABTS**			
	**D0**	**D30**	**D90**	**D150**	**D0**	**D30**	**D90**	**D150**
C-A	327.61 ± 1.69 ^A^	236.39 ± 13.18 ^a,B^	96.10 ± 5.13 ^C^	72.44 ± 3.69 ^b,D^	0.23 ± 0.06 ^A^	0.20 ± 0.02 ^A^	0.06 ± 0.01 ^b,B^	0.04 ± 0.01 ^b,B^
C-Ar	327.61 ± 1.69 ^A^	204.59 ± 7.42 ^b,B^	96.91 ± 4.34 ^C^	78.24 ± 3.31 ^a,D^	0.23 ± 0.06 ^A^	0.22 ± 0.04 ^A^	0.10 ± 0.01 ^a,B^	0.05 ± 0.01 ^ab,B^
C-Ar-BB	327.61 ± 1.69 ^A^	239.20 ± 12.88 ^a,B^	97.42 ± 4.14 ^C^	83.04 ± 1.40 ^a,C^	0.23 ± 0.06 ^A^	0.23 ± 0.03 ^A^	0.10 ± 0.01 ^a,B^	0.05 ± 0.01 ^a,B^
C-Ar-SL	327.61 ± 1.69 ^A^	234.31 ± 3.72 ^a,B^	98.17 ± 4.39 ^C^	81.62 ± 1.70 ^a,D^	0.23 ± 0.06 ^A^	0.21 ± 0.02 ^A^	0.09 ± 0.01 ^a,B^	0.05 ± 0.01 ^ab,B^
T-A	299.89 ± 3.34 ^A^	217.67 ± 10.99 ^a,B^	115.62 ± 7.13 ^C^	102.37 ± 3.41 ^C^	0.23 ± 0.07 ^A^	0.25 ± 0.03 ^A^	0.10 ± 0.01 ^b,B^	0.07 ± 0.01 ^B^
T-Ar	299.89 ± 3.34 ^A^	244.78 ± 2.53 ^a,B^	122.96 ± 2.51 ^C^	110.01 ± 4.94 ^D^	0.23 ± 0.07 ^A^	0.27 ± 0.04 ^A^	0.12 ± 0.01 ^a,B^	0.08 ± 0.01 ^B^
T-Ar-BB	299.89 ± 3.34 ^A^	213.31 ± 5.12 ^a,B^	116.58 ± 8.08 ^C^	107.10 ± 0.35 ^C^	0.23 ± 0.07 ^A^	0.23 ± 0.05 ^A^	0.12 ± 0.01 ^a,B^	0.08 ± 0.01 ^B^
T-Ar-SL	299.89 ± 3.34 ^A^	214.56 ± 9.80 ^b,B^	120.79 ± 3.70 ^C^	107.12 ± 4.46 ^D^	0.23 ± 0.07 ^A^	0.22 ± 0.02 ^A^	0.12 ± 0.01 ^ab,B^	0.08 ± 0.01 ^B^
**(B) Phenols/ABTS**	**Malaxation Process**			**Packaging Conditions**		**Malaxation Process × Packaging Conditions**		
D0	***/n.s.			n.s./n.s.		n.s./n.s.		
D30	**/*			**/n.s.		***/n.s.		
D90	***/***			n.s./***		n.s./n.s.		
D150	***/***			**/*		n.s./n.s.		

Lowercase upper letters evidenced significant differences among the different packaging conditions of control and test samples. Uppercase upper letters evidenced significant differences among the different storage times. LSR, * *p* < 0.05; ** *p* < 0.01; *** *p* < 0.0001; n.s.: not significative.

**Table 8:** (**A**) Pigment content progressions during the storage of the control and the test oil packaged in alternative conditions. (**B**) Two-way analysis of variance performed to evaluate the influence of the malaxation process, packaging conditions, and their interaction on the pigment concentration of the samples during the storage.

**(A) Samples**	**Carotenoids**				**Chlorophylls**			
	**D0**	**D30**	**D90**	**D150**	**D0**	**D30**	**D90**	**D150**
C-A	3.10 ± 0.07 ^A^	2.48 ± 0.01 ^B^	0.71 ± 0.00 ^b,C^	0.58 ± 0.00 ^c,D^	6.36 ± 0.0 ^A^	4.31 ± 0.02 ^B^	3.04 ± 0.00 ^b,C^	2.06 ± 0.00 ^D^
C-Ar	3.10 ± 0.07 ^A^	2.02 ± 0.08 ^B^	0.79 ± 0.01 ^b,C^	0.60 ± 0.00 ^b,D^	6.36 ± 0.07 ^A^	4.77 ± 0.0602 ^B^	3.32 ± 0.00 ^a,C^	2.18 ± 0.01 ^D^
C-Ar-BB	3.10 ± 0.07 ^A^	2.57 ± 0.01 ^B^	2.08 ± 0.01 ^a,C^	0.66 ± 0.00 ^a,D^	6.36 ± 0.07 ^A^	4.35 ± 0.0602 ^B^	3.20 ± 0.00 ^ab,C^	2.09 ± 0.00 ^D^
C-Ar-SL	3.10 ± 0.07 ^A^	2.51 ± 0.02 ^B^	2.01 ± 0.04 ^b,C^	0.64 ± 0.00 ^a,D^	6.36 ± 0.07 ^A^	4.29 ± 0.0002 ^B^	3.05 ± 0.01 ^b,C^	2.22 ± 0.00 ^D^
T-A	3.04 ± 0.01 ^A^	2.19 ± 0.04 ^B^	0.81 ± 0.01 ^a,C^	0.71 ± 0.01 ^b,D^	5.77 ± 0.19 ^A^	5.41 ± 0.02 ^A^	3.38 ± 0.00 ^B^	2.59 ± 0.00 ^c,C^
T-Ar	3.04 ± 0.01 ^A^	2.38 ± 0.03 ^B^	0.84 ± 0.01 ^b,C^	0.75 ± 0.00 ^ab,D^	5.77 ± 0.19 ^A^	5.84 ± 0.02 ^A^	3.49 ± 0.00 ^B^	2.91 ± 0.00 ^a,C^
T-Ar-BB	3.04 ± 0.01 ^A^	2.61 ± 0.06 ^B^	2.30 ± 0.03 ^a,C^	0.73 ± 0.01 ^ab,D^	5.77 ± 0.19 ^A^	4.46 ± 0.00 ^A^	3.30 ± 0.00 ^B^	2.81 ± 0.00 ^b,C^
T-Ar-SL	3.04 ± 0.01 ^A^	2.70 ± 0.05 ^B^	2.35 ± 0.02 ^b,C^	0.76 ± 0.00 ^a,D^	5.77 ± 0.19 ^A^	4.40 ± 0.03 ^A^	3.2 ± 0.03 ^B^	2.74 ± 0.00 ^b,C^
**(B) Carotenoids/Chlorophylls**	**Malaxation Process**			**Packaging Conditions**		**Malaxation Process × Packaging Conditions**		
T0	*/***			n.s./n.s.		n.s./n.s.		
T30	n.s./***			n.s./***		n.s./***		
T90	***/*			**/*		**/n.s.		
T150	***/***			***/***		**/**		

Lowercase upper letters evidenced significant differences among the different packaging conditions of control and test samples. Uppercase upper letters evidenced significant differences among the different storage times. LSR, * *p* < 0.05; ** *p* < 0.01; *** *p* < 0.0001; n.s.: not significative.

## Data Availability

The original contributions presented in the study are included in the article, further inquiries can be directed to the corresponding author.
